# Comprehensive analysis for cellular senescence-related immunogenic characteristics and immunotherapy prediction of acute myeloid leukemia

**DOI:** 10.3389/fphar.2022.987398

**Published:** 2022-09-26

**Authors:** Yan Mao, Jinwen Xu, Xuejiao Xu, Jiayun Qiu, Zhengyun Hu, Feng Jiang, Guoping Zhou

**Affiliations:** ^1^ Department of Pediatrics, The First Affiliated Hospital of Nanjing Medical University, Nanjing, China; ^2^ Department of Pediatric Nephrology, Wuxi Children’s Hospital Affiliated to Nanjing Medical University, Wuxi, China; ^3^ Department of Pediatrics, Shanghai Songjiang District Central Hospital, Shanghai, China; ^4^ Department of Neonatology, Obstetrics and Gynecology Hospital of Fudan University, Shanghai, China

**Keywords:** cellular senescence, acute myeloid leukemia, senescence-associated secretory phenotype, tumor microenvironment, tumor mutation burden, immunotherapy

## Abstract

In malignancies, cellular senescence is critical for carcinogenesis, development, and immunological regulation. Patients with acute myeloid leukemia (AML) have not investigated a reliable cellular senescence-associated profile and its significance in outcomes and therapeutic response. Cellular senescence-related genes were acquired from the CellAge database, while AML data were obtained from the GEO and TCGA databases. The TCGA-AML group served as a training set to construct a prognostic risk score signature, while the GSE71014 set was used as a testing set to validate the accuracy of the signature. Through exploring the expression profiles of cellular senescence-related genes (SRGs) in AML patients, we used Lasso and Cox regression analysis to establish the SRG-based signature (SRGS), which was validated as an independent prognostic predictor for AML patients via clinical correlation. Survival analysis showed that AML patients in the low-risk score group had a longer survival time. Tumor immune infiltration and functional enrichment analysis demonstrated that AML patients with low-risk scores had higher immune infiltration and active immune-related pathways. Meanwhile, drug sensitivity analysis and the TIDE algorithm showed that the low-risk score group was more susceptible to chemotherapy and immunotherapy. Cell line analysis *in vitro* further confirmed that the SRGs in the proposed signature played roles in the susceptibility to cytarabine and YM155. Our results indicated that SRGS, which regulates the immunological microenvironment, is a reliable predictor of the clinical outcome and immunotherapeutic response in AML.

## 1 Introduction

Acute myeloid leukemia (AML) is a variable disease that arises from the malignant clonal expansion of myeloid progenitor cells, which are mainly located in the bone marrow and peripheral blood (Jiang et al., 2021a). It is the most prevalent form of adult leukemia, accounting for about 2.5% of new cases and about 3.1% of new deaths globally in 2020. This data ranks AML among the top causes of cancer-related mortality (Sung et al., 2021). Chemotherapy, as well as stem-cell transplants, remain the principal treatment options for AML patients at present (Jiang et al., 2021b). Over the last few decades, breakthroughs in our knowledge of the biology of AML, along with the aggressive consolidation of chemotherapy regimens, improved supportive care, and better stem-cell transplantation methods (Coombs et al., 2016), contribute to higher overall survival (OS) time for AML patients (Jiang et al., 2021c). However, the overall results remain dismal. Consequently, it is vital for us to investigate appropriate treatment techniques for AML patients in order to enhance their prognosis.

Cellular senescence is an essential aspect of aging (Campisi, 2013) and a relationship between aging and cancers (Liu et al., 2020; Partridge et al., 2018). However, the relationship between senescence and cancers is still complicated and poorly understood. Previous research has shown that senescence is a double-edged sword in cancer development. First, senescence maintains tissue homeostasis and inhibits the genesis of tumors when senescent cells enter and suffer permanent cell cycle arrest (Kumari et al., 2021; Perez-Mancera et al., 2014). In early carcinogenesis, senescence works as a barrier to tumor formation when it is followed by immune clearance and tissue remodeling (Ray and Yung., 2018; Xue et al., 2007). Second, cellular senescence may have negative consequences if the immune system does not eliminate senescent cells and the senescent cells accumulate. This buildup leads to the development of senescence-associated secretory phenotype (SASP), resulting in both aging and tumor formation (Cuollo et al., 2020) by triggering the release of growth factors, cytokines, extracellular matrix enzymes, and extracellular matrix components (Liu et al., 2016; Lopes-Paciencia et al., 2019; Menicacci et al., 2017). To formulate innovative therapy paradigms for malignancies, a better knowledge of the influence of senescence on tumor immunology in relation to invasion and development is necessary. Recent research indicates that tumor cells might experience senescence as an evolutionary process, which involves both tumor intrinsic traits and external immunological load (Berben et al., 2021; Kumari and Jat., 2021; Zhou et al., 2021). Notably, the negative consequences of SASP outweigh its positive features (Cuollo et al., 2020). Thereby, we hypothesized that along with the accumulation of senescent cells, SASP remodels the tumor microenvironment (TME) through recruiting immunosuppressive cells, thereby promoting the evasion of tumor cells from immunosurveillance and leading to poor clinical outcomes in AML.

In order to thoroughly analyze the relationships between cellular senescence and AML patients’ prognosis, we developed a new signature according to cellular senescence-related genes (SRGs). We then investigated the potential value of serving as prognostic and immunotherapy response biomarkers. According to the SRG-related signature (SRGS), the interactions between risk groupings and immunological checkpoints, as well as immune cell infiltration, were then carefully investigated. Additional investigation of the processes revealed that cellular senescence in AML influenced the TME through SASP. In a word, this work gave new insights into the potential regulatory mechanisms related to cellular senescence and relevant immunotherapeutic treatments for AML.

## 2 Methods

### 2.1 Data acquisition and processing

Clinical information and transcriptional profiles of AML patients were from The Cancer Genome Atlas (TCGA, https://portal.gdc.cancer.gov) and the Gene Expression Omnibus (GEO, http://www.ncbi.nlm.nih.gov/geo). The TCGA-AML cohort (Weinstein et al., 2013) containing 129 samples was enrolled as the training set for constructing a prognostic risk model, while the GSE71014 cohort (Wang et al., 2021; Chuang et al., 2015; Lee et al., 2017) containing 104 AML samples was enrolled as testing set for validation. Since the samples in TCGA-AML were all tumor samples, we downloaded the whole blood cohorts from GTEx from the UCSC Xena database as control samples. The batch effect of the expression profiles from the standardized RNA-seq data of TCGA and GTEx was removed through the “sva” package to eliminate the influence between the two datasets.

### 2.2 Development and validation of the cellular senescence-related signature

The genes associated with cellular senescence were obtained from CellAge (Avelar et al., 2020) (https://genomics.senescence.info/cells/). A total of 279 SRGs (Supplementary Table S1) were included in this study. We first screened differentially expressed SRGs (DESRGs) between normal and AML samples according to the SRGs’ levels. Univariate Cox analysis was carried out to identify SRGs with prognostic values. The LASSO Cox regression analysis was carried out to construct the model for predicting the survival of AML patients. Through tenfold cross-validations, the best values for the penalty parameter lambda were identified. On the basis of the median risk score derived by SRGS, patients in the training or testing cohorts were separated into high- and low-risk groups, and SRGS's performance was then assessed. To investigate the independence of the signature in prediction, we carried out univariate and multivariate Cox analyses for the variables in clinic and SRGS in the TCGA-AML set. The chosen clinical variables mainly included gender, peripheral blood (PB) blasts percent (PB-blast), bone marrow (BM) blast percent (BM_blast), hemoglobin, white blood cell (WBC), and platelets levels, all of which have been found important for the prognosis of AML patients (McQuilten et al., 2022; Acharya et al., 2018; Just et al., 2019; Liu et al., 2019; Ogawa et al., 2018; Short et al., 2016; Ustun et al., 2021).

### 2.3 Functional enrichment pathway analysis and correlation between immune cell infiltration and cellular senescence-related signature

Estimation of immune infiltration was carried out using the EPIC algorithm (Racle et al., 2017), XCELL algorithm (Aran et al., 2017), CIBERSORT algorithm (Newman et al., 2015), MCPCOUNTER algorithm (Petitprez et al., 2020), and QUANTISEQ algorithm (Finotello et al., 2019) in R software. Tumor-infiltrating lymphocyte features were also carried out to estimate the immune cell infiltration using the gsva algorithm. In addition, we conducted the Pearson correlation analysis to clarify the relationship between the built SRGS and immune cell infiltration. We further utilized the ESTIMATE algorithm to explore the infiltration degree of tumor and normal cells to determine the levels of StromalScore, ImmuneScore, and EstimateScore (Yoshihara et al., 2013). Besides, tumor stem cell features, which were extracted from the transcriptome and epigenetics of TCGA-AML samples, were carried out to estimate the stem cell-like features (Dib et al., 2017).

### 2.4 Assessment of cellular senescence-related signature and response to immune checkpoint inhibitors and chemotherapy drugs

Tumor Immune Dysfunction and Exclusion (TIDE) (Fu et al., 2020; Jiang et al., 2018), http://tide.dfci.harvard.edu/) is developed for assessing the immune evasion mechanisms. Hence, TIDE is often used as another robust biomarker for predicting immunotherapy response. While a higher TIDE score usually suggests a lower response rate for tumor cells to immunotherapy. The tumor mutation burden (TMB) for each AML patient was measured by the nonsynonymous mutation numbers per mega-base. The pRRophetic package (Geeleher et al., 2014) in R software was utilized for predicting the half-maximal inhibitory concentration (IC_50_) of cytarabine and YM155 in each TCGA-AML sample. In addition, the Pearson correlation analysis was carried out to explore the positive relationships between the prognostic SRGS and sensitivity to cytarabine or YM155.

### 2.5 *In vitro* experiments

AML cell line (U937) was obtained from the American Type Culture Collection (ATCC, USA). All cells were grown in an incubator at 37°C and 5% CO_2_ in a humid environment. Cells were grown in RPMI-1640 media supplemented with 10% fetal bovine serum, 100 IU/mL penicillin, and 100 mg/mL streptomycin, respectively. Cytarabine and YM155 were acquired from Selleck Chemicals (United States). Using the Cell Counting Kit-8 (CCK-8) test (Dojindo, Japan), the viability of cells was determined. Twenty thousand cells per well were seeded in a 96-well plate and incubated at 37°C in a humidified cell incubator containing 5% CO_2_. After 48 h of exposure to the chemical at the prescribed concentrations, the CCK-8 reagent was added, and incubation continued for an additional 2 h. Optical density (OD) was determined using the Tecan Spark TM10M at 450 nm (TECAN, Switzerland). For Western Blot analysis, cells were centrifuged at 1000 rpm for 5 min after 48 h of exposure to the drug at various doses. This was followed by a 0.5-hour resuspension in protease and phosphatase inhibitor-containing RIPA lysis buffer (PHYGENE, China). Before being centrifuged at 12000 rpm at 4°C for 15 min, the lysates were subjected to vortex and sonication in a bath of cold water for 5 min on high with 30-second intervals and 1 min off. After determining the protein concentration, the protein was combined with loading buffer and boiled for 10 min at 100°C, followed by electrophoretic separation on a 10% SDS-PAGE and transferred to PVDF membranes. The membrane was then treated with 5% skim milk powder for 2 h at room temperature to eliminate nonspecific interaction, followed by overnight incubation with antibodies against GAPDH (Proteintech, China), BAX (CST, United States), and BCL-2 (CST, United States) at 4°C, and subsequent incubations in secondary antibodies (CST, United States), prior to visualization with enhanced chemiluminescence (ECL) western (GBCBIO Technologies, China). For the detection of mRNA levels, we used SYBR green I dye from Takara (Dalian, China) and the ABI 7500 real-time PCR machine (Applied Biosystems, United States). Sequences of primers were designed in PrimerBank (http://pga.mgh.harvard.edu/primerbank/). We used the following primers: KDM5B (Primer Bank ID: 57242795c1), SMURF2 (Primer Bank ID: 56550041c1), MAP4K1 (Primer Bank ID: 110611904c1), G6PD (Primer Bank ID: 108773794c2), CDK18 (Primer Bank ID: 262527294c1), SOCS1 (Primer Bank ID: 4507232c1), ETS2 (Primer Bank ID: 372466581c3), AKR1B1 (Primer Bank ID: 24497579c2), and GAPDH (Primer Bank ID: 378404907c2).

### 2.6 Statistical analysis

All the data analysis and the graph generation in this study were carried out in R version 3.5.1, SPSS version 25.0, and GraphPad Prism version 8.0. Unpaired Student’s t-test was applied for comparisons of two groups to analyze the statistical significance. The Kaplan-Meier survival analyses for the OS of AML patients were performed using the R package named “survminer”. Receiver operating characteristic (ROC) curves for survival evaluated the predictive efficacy of the SRGS (Blanche et al., 2013). Univariate and multivariate Cox analyses were carried out to explore the relationship between OS and SRGS scores, as well as clinical characteristics. *p* < 0.05 was considered statistically significant.

## 3 Results

### 3.1 Identification of DESRGs with prognostic value

**FIGURE 1:**
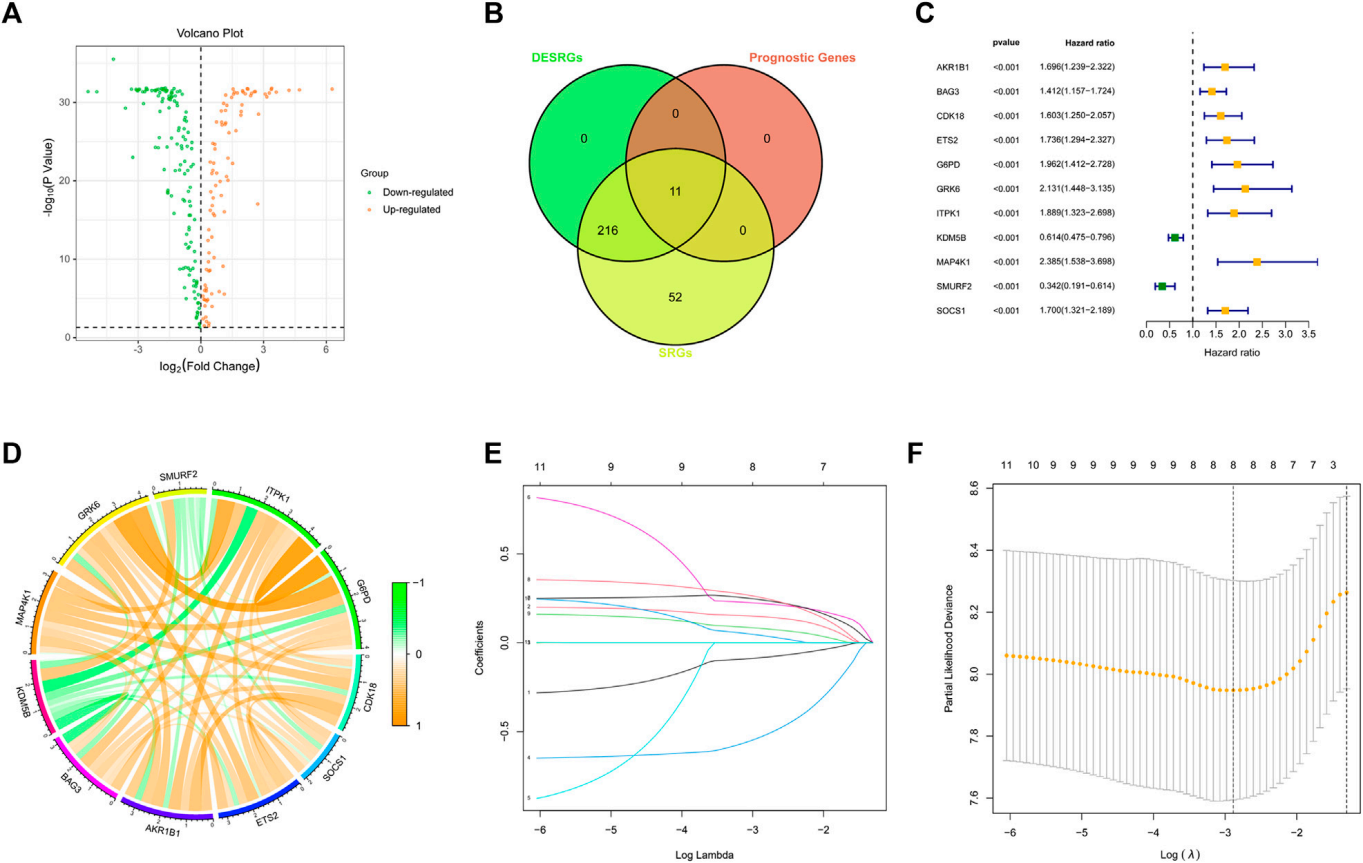
Identification of DESRGs with prognostic value. **(A)** Volcano plot of SRGs in the TCGA dataset. **(B)** Eleven overlapping genes in SRGs, DESRGs, and prognostic genes. **(C)** Results of the univariate Cox analysis based on the identified candidate 11 SRGs. **(D)** Correlation network of the 11 candidate SRGs. **(E)** LASSO analysis on the 11 candidate SRGs. **(F)** Cross-validation in the LASSO regression.

A total of 278 SRGs were compared between AML samples from TCGA and normal samples from GTEx to characterize their expression in AML. We identified a total of 227 DESRGs (Figure 1A). Next, a univariate Cox analysis was initially carried out on the 278 SRGs to identify the ones associated with the AML patients’ OS. A total of 11 SRGs were then found significantly related to OS, all of which overlapped with the DESRGs and were considered risk factors (Figure 1B). Nine of the 11 genes (AKR1B1, BAG3, CDK18, ETS2, G6PD, GRK6, ITPK1, KDM5B, MAP4K1, SMURF2, and SOCS1) were upregulated, while the other two genes were downregulated in AML (Figure 1C). Besides, 11 prognostic DESRGs were correlated with each other closely (Figure 1D).

### 3.2 Development and validation of the cellular senescence-related signature in acute myeloid leukemia

**FIGURE 2:**
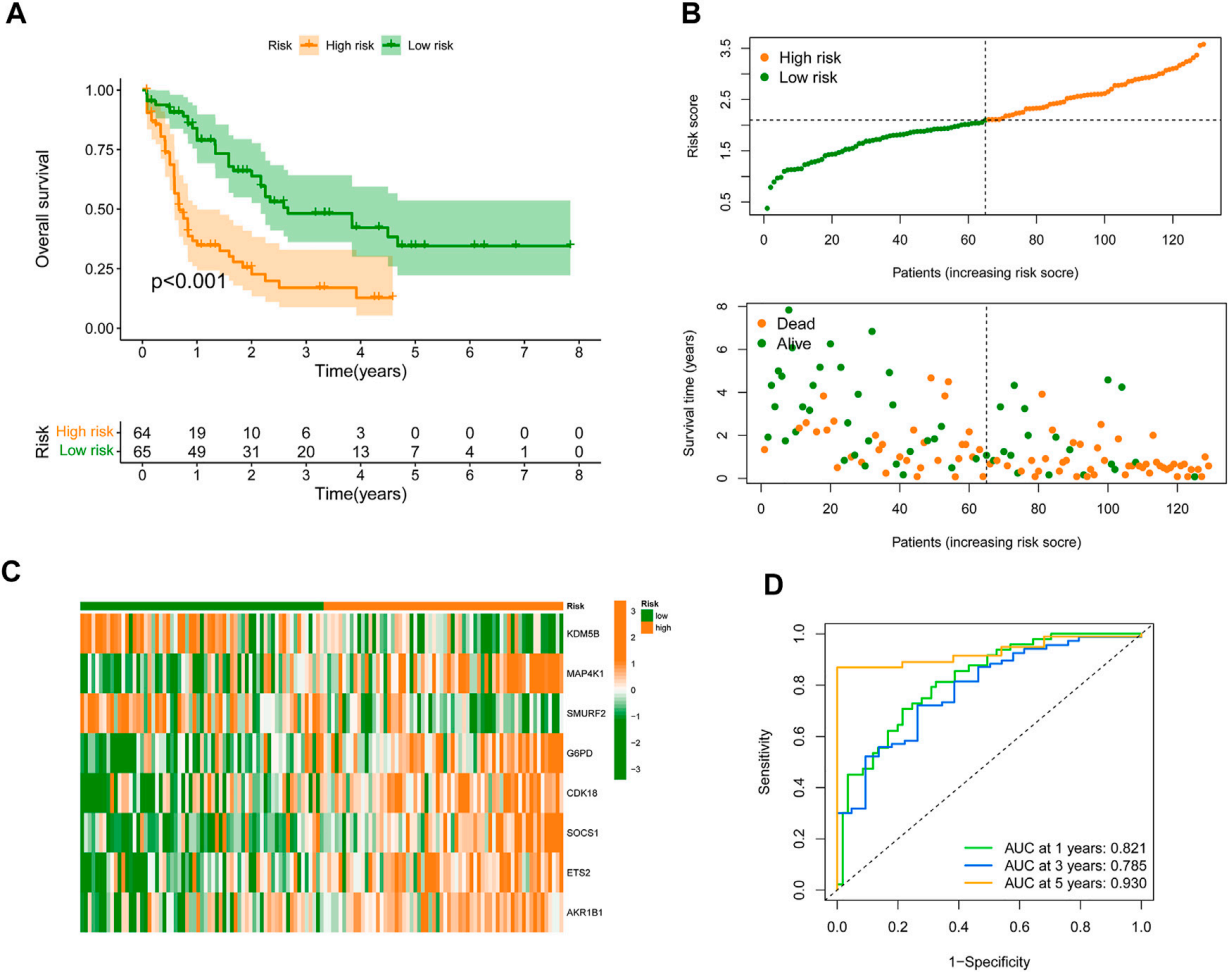
Development of SRGS in TCGA-AML cohort. **(A)** Kaplan-Meier curve analysis of the OS in the TCGA-AML patients. **(B)** The risk score distribution and survival status scatter plots of TCGA-AML patients. **(C)** Heatmap of the eight signature genes in the risk groups. **(D)** Time-dependent ROC analysis of the SRGS.

To construct an SRGS for the survival prediction in AML, the 11 OS-associated SRGs were analyzed using LASSO Cox analysis. An 8-gene SRGS was then built (Figures 1E,F). A score formula was established as followed: risk score = (−0.0838 × KDM5B level) + (0.1467 × MAP4K1 level) + (−0.5299 × SMURF2 level) + (0.2210 × G6PD level) + (0.2415 × CDK18 level) + (0.2543 × SOCS1 level) + (0.0803 × ETS2 level) + (0.0411 × AKR1B1 level). The score of each AML patient from the TCGA was then calculated. AML patients were stratified into different risk groups using the median value to serve as the cutoff. Kaplan-Meier survival analysis results in Figure 2A demonstrated that AML patients in the high-risk group had shorter OS time. The distribution of SRGS scores and the survival status of samples were presented in Figure 2B. The heatmap exhibited the chosen eight genes’ expression profiles in two risk groups (Figure 2C). The areas under the curve (AUCs) for 1-, 3-, and 5-years OS were respectively 0.821, 0.785, and 0.930 (Figure 2D). These results indicated that the prognostic SRGS could classify AML patients with different OS.

**FIGURE 3:**
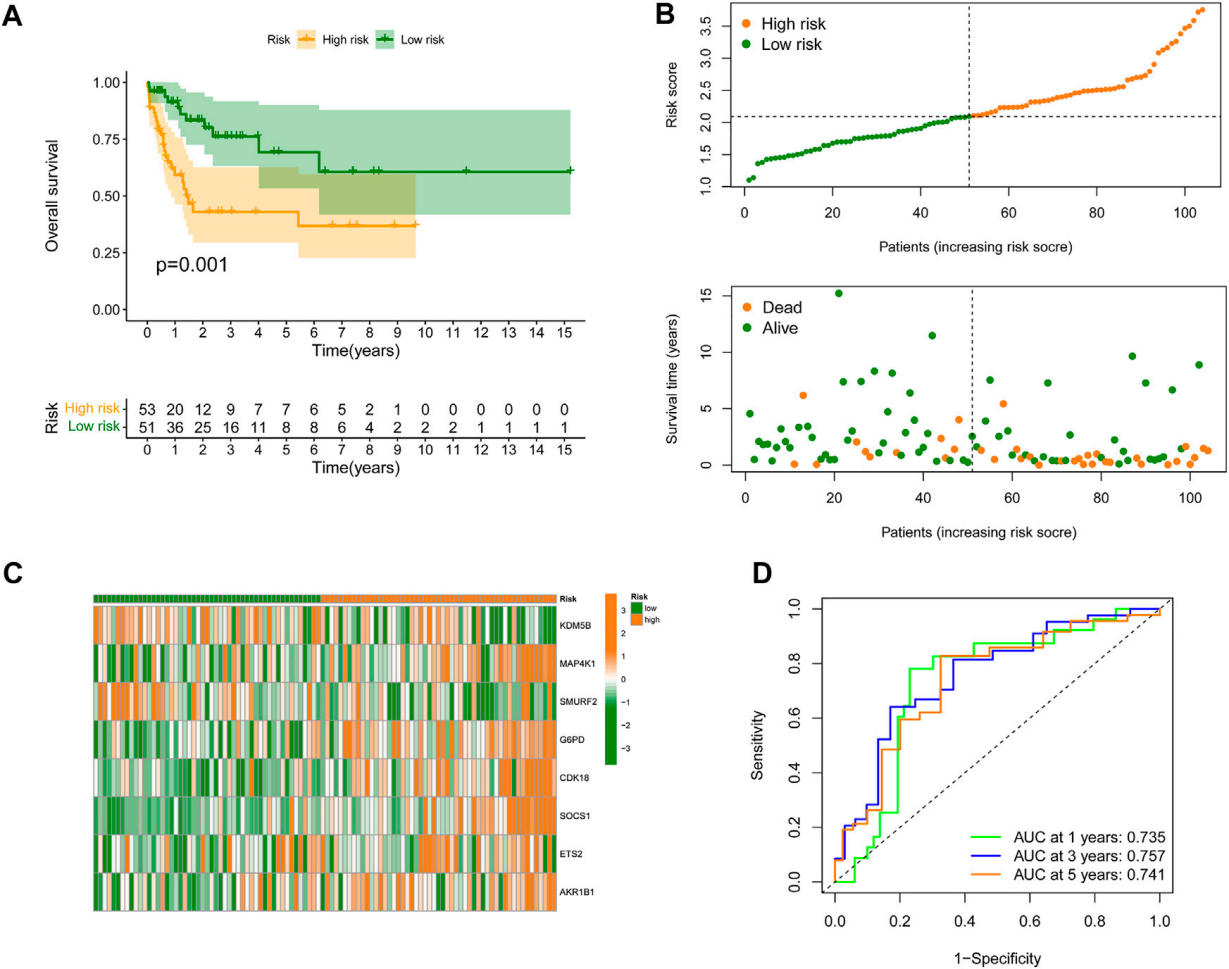
Validation of the SRGS in an external GEO cohort. **(A)** Kaplan-Meier curve analysis of OS in AML patients from the GSE71014 dataset. **(B)** The risk score distribution and survival status scatter plots. **(C)** Heatmap of the eight genes in the proposed SRGS in two risk groups from GSE71014 dataset. **(D)** Time-dependent ROC analysis of the SRGS.

To further validate the predictive value of the constructed SRGS, an external AML dataset named GSE71014 from the GEO database was enrolled. Kaplan-Meier survival analysis in Figure 3A also showed that patients with higher SRGS scores had shorter OS time. The distribution of score, the survival status, and genes’ expression profiles in Figures 3B,C had the same trend as those in Figures 2B,C. The AUC values for 1-, 3-, and 5-years OS in GSE71014 were all greater than 0.7 (Figure 3D), confirming that SRGS was a good predictive factor for AML.

### 3.3 Independent prognostic value of cellular senescence-related signature and construction of clinical nomogram

**FIGURE 4:**
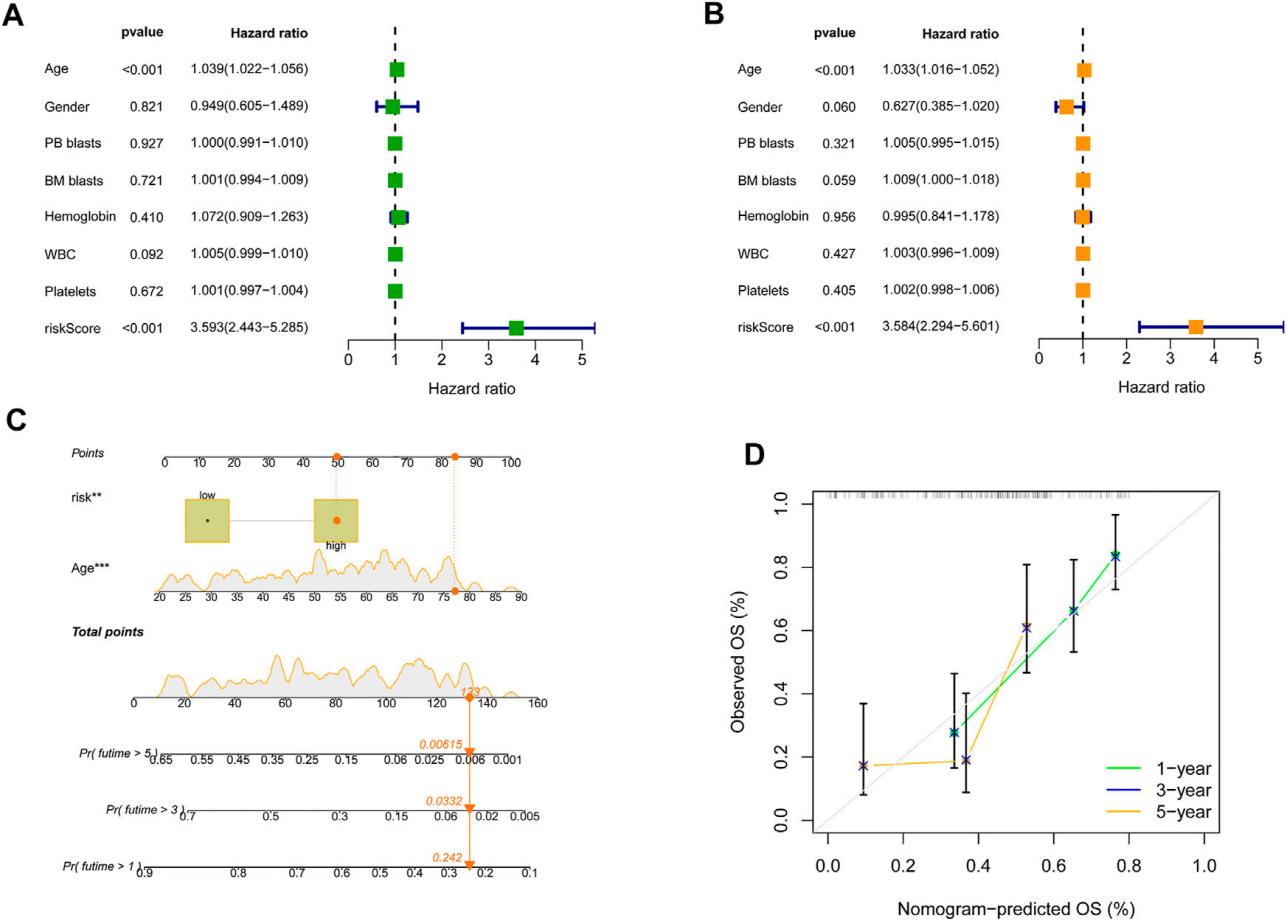
The independence of predictive value of the SRGS and construction of the clinical nomogram in TCGA-AML cohort. **(A)** Results of the univariate Cox analysis based on OS-related factors. **(B)** Results of the multivariate Cox analysis based on OS-related factors. **(C)** Nomogram constructed in conjunction with the SRGS and clinical characterization. **(D)** The calibration plot of the nomogram.

Univariate and multivariate Cox analyses for the variables in clinic and SRGS were carried out to determine the independence of SRGS’s prognostic value for OS of AML patients. As shown in Figure 4A, the scores of SRGS for samples in the TCGA-AML cohort were significantly related to the patients’ clinical OS. The results of multivariate Cox analysis in Figure 4B showed that the SRGS remained an independent predictor. Apart from the SRGS, we found age could also serve as an independent predictor, while the other chosen clinical variables showed no significant difference (*p* > 0.05; Figures 4A,B). Combining the above factors, we then established a nomogram to help broaden the application of the SRGS in the clinic (Figure 4C). Every AML patient from the TCGA cohort was assigned a value by adding all the prognostic parameters’ points. And patients with higher total points owned worse clinical outcomes. The calibration plot in Figure 4D showed that the established clinical nomogram an excellent performance.

### 3.4 Analyses of enrichment pathways and the alterations in SASP and immune cell infiltration in acute myeloid leukemia

**FIGURE 5:**
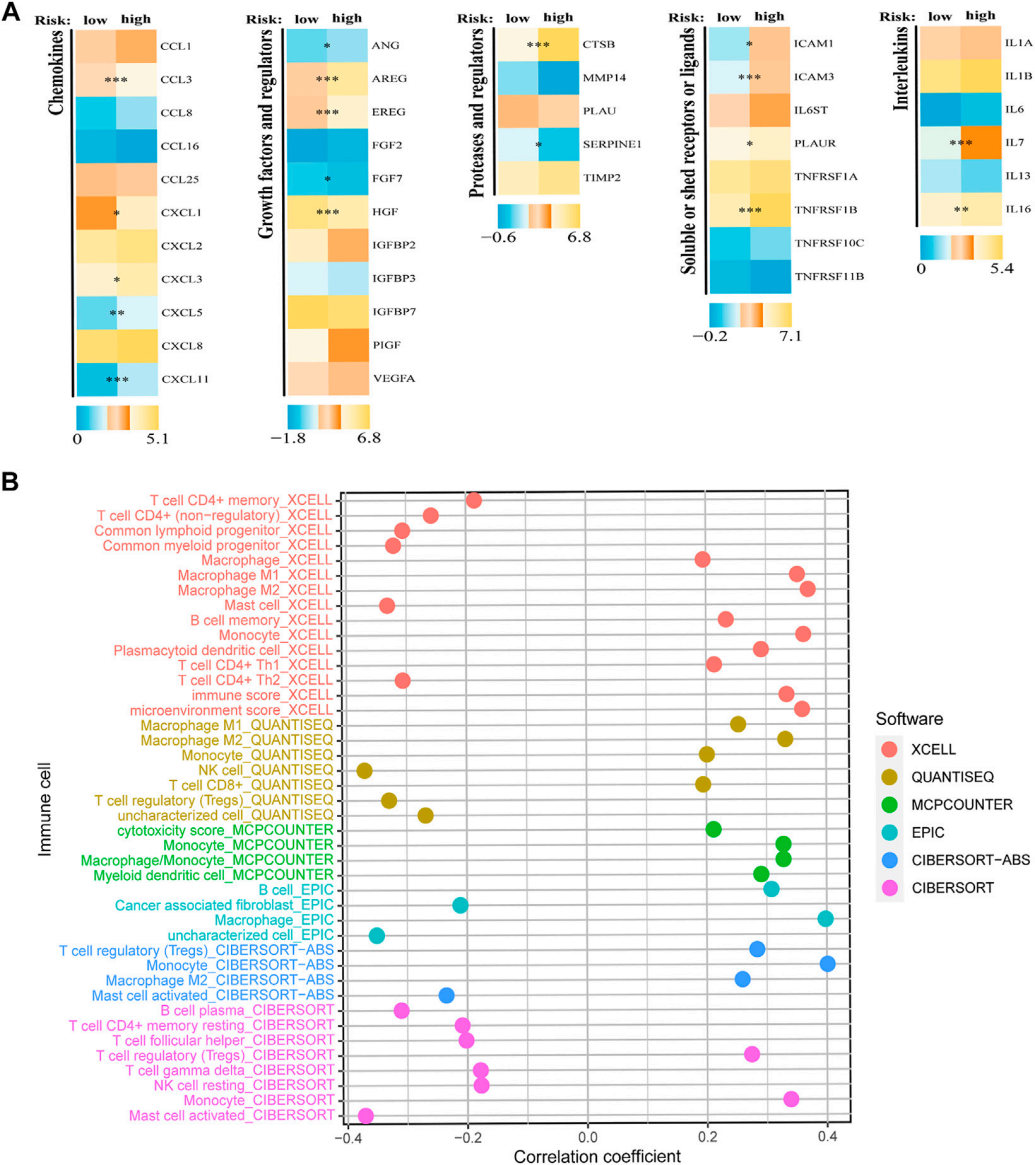
Analyses of SASP and immune cell infiltration levels. **(A)** Expression of different types of SRGS-associated secretory phenotype factors between two risk groups. **(B)** Correlation analysis between SRGS and immune cell infiltration abundance.

The evaluation above confirmed the predictive value of the constructed SRGS, prompting us to further explore the possible mechanism. Gene set variation analysis (GSVA) was done to elucidate the biological functions of various risk groups in the tumorigenicity and progression of AML (Supplementary Figure S1). AML samples from the lower risk group showed heightened activities of the MYC targets pathway. While, the majority of high expressed genes in the high-risk AML group were enriched in the PPAR signaling pathway, chemokine signaling pathway, and calcium signaling pathway. SASP demonstrates that senescent cells release a vast number of secretory proteins, which may promote alterations in the TME (Jiang et al., 2022), hence encouraging tumor recurrence and progression (Green, 2008; Kuilman et al., 2008; Li et al., 2012; Richardson et al., 2021). In the AML group with higher SRGS scores, a number of SASP types were overexpressed, per our findings (Figure 5A). Chemokines (CCL3, CXCL1, CXCL3, CXCL5, and CXCL11), interleukins (IL-7, and IL-16), growth factors and regulators (EREG, ANG, AREG, FGF7, and HGF), soluble or shed receptors or ligands (TNFRSF11B, PLAUR, ICAM3, and ICAM1), and proteases and regulators (CTSB, and SERPINE1) were significantly upregulated. These results confirmed higher SASP levels in high-risk AML patients.

Some upregulated SASPs possess immunosuppressive properties (Lamano et al., 2019; Sharma et al., 2017). Consequently, we anticipated that individuals with high SRGS scores and elevated SASP levels could have an immunosuppressive phenotype through SASP. RNAseq-derived infiltrating immune cell populations were calculated in order to define the immunological landscape associated with SRGS. We discovered that groups stratified by SRGS had unique patterns of immunological infiltrates. The infiltration levels of activated mast cells, follicular T helper cells, NK cells, resting mast cells, cancer-associated fibroblasts, and regulatory T cells were negatively correlated to SRGS scores. In contrast, higher SRGS scores indicated greater abundances of the M2 macrophages, B cells, monocytes, myeloid dendritic cells, and M1 macrophages (Figure 5B).

### 3.5 Correlation of chosen immune checkpoints and the risk score and their impact on clinical outcome of TCGA-acute myeloid leukemia patients

**FIGURE 6:**
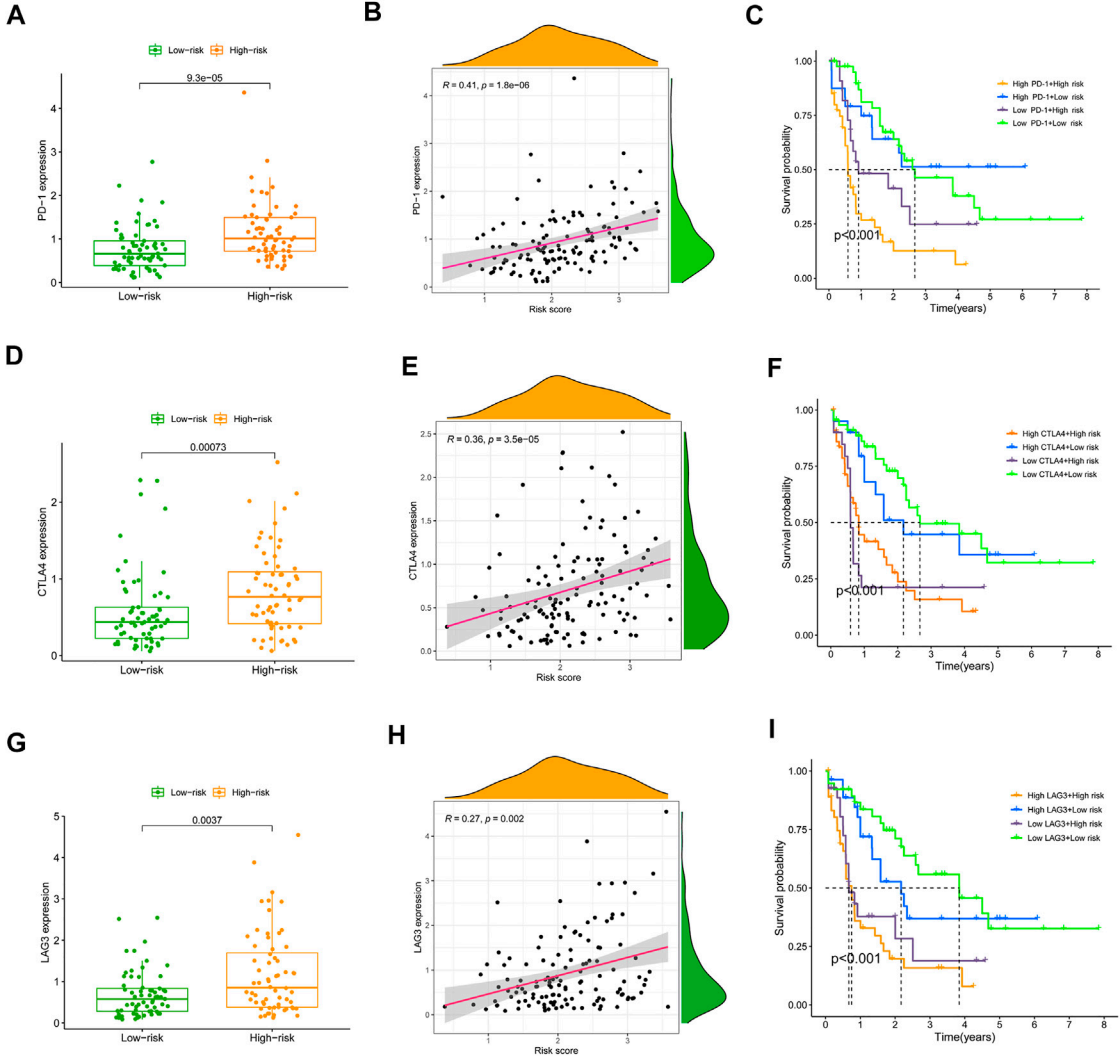
Correlation of chosen immune checkpoints and the SRGS score and their impact on clinical outcome of TCGA-AML patients. **(A,D,G)** Comparison of the PD-1, CTLA-4 or LAG3 expression level between different AML risk groups. **(B,E,H)** Correlation between SRGS score and the PD-1, CTLA-4 or LAG3 expression level. **(C,F,I)** Kaplan-Meier survival analyses of OS in the four groups grouped by the SRGS and the level of PD-1, CTLA-4 or LAG3.

Previous research has shown the significance of immune checkpoint genes in regulating immune infiltration (Juneja et al., 2017; Kumagai et al., 2020). To further study the complicated interplay between immune checkpoints and the established SRGS, we evaluated their expression patterns across SRGS-based groups. As shown in Figures 6A,D, AML patients with higher SRGS scores expressed higher levels of two chosen immune checkpoint genes (PD-1 and CTLA4) in the TCGA cohort. Another checkpoint, LAG3, which is considered an exhausted T cell marker, also showed an overexpression trend in the group with higher SRGS scores, suggesting that the SRGS owned the ability to identify immune dysfunction (Figure 6G). Meanwhile, the expression levels of the three chosen checkpoint genes showed positive correlations to the SRGS scores (Figures 6B,E,H). Then, we analyzed SRGS in conjunction with immune checkpoint expression to determine if SRGS affects the OS of AML patients with comparable checkpoint genes’ expression. Survival analysis was carried out on four groups stratified by SRGS and immune checkpoint gene expression. Figure 6C illustrated that those individuals with low PD-1 expression levels and low SRGS scores had a longer OS than those with low PD-1 expression levels and high SRGS scores. In individuals with strong PD-1 expression levels, a lower risk score indicated a survival rate that was significantly improved. In the TCGA-AML cohort, similar survival trends were identified across the four AML patient groups stratified by the SRGS scores and CTLA4 (Figure 6F) or LAG3 (Figure 6I) expression.

### 3.6 Correlation between the cellular senescence-related signature and tumor microenvironment

**FIGURE 7:**
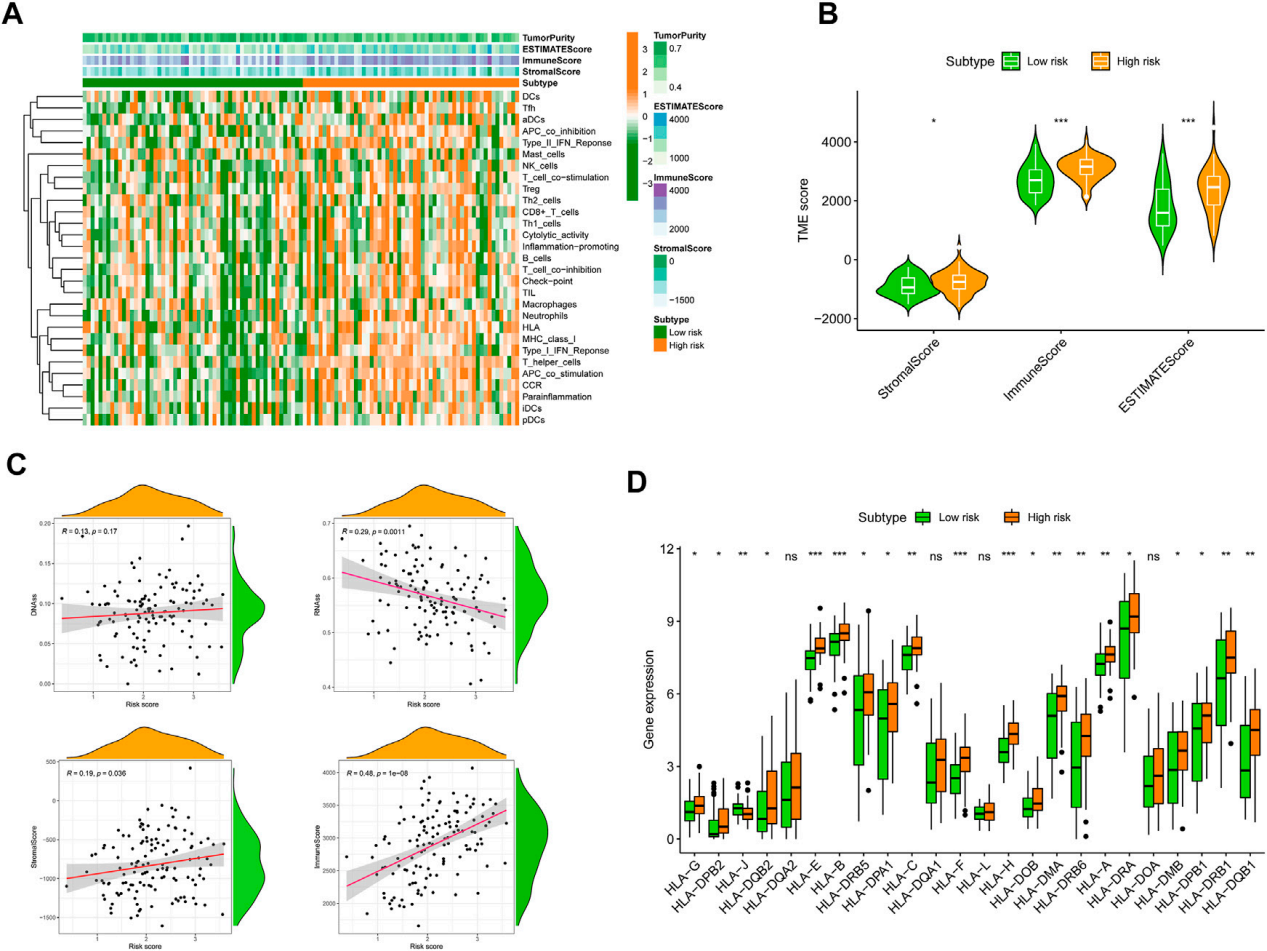
Correlation between SRGS and TME. **(A)** Landscape of the immune characteristics and TME. **(B)** Correlations between SRGS score and TME score. **(C)** The relationships between SRGS scores and DNAss, RNAss, Stromal Scores, and Immune Scores. **(D)** Comparison of HLA gene expression levels between two risk AML groups.

To completely characterize the immunological aspects of AML, samples from the TCGA-AML cohort were evaluated. Based on the SRGS scores and hierarchical clustering method, all AML samples were grouped cleanly into two groups (Figure 7A). The features of the TME between the two AML groups were discovered based on the findings of ESTIMATE. We discovered that the groups with the higher SRGS scores had higher EstimateScore, ImmuneScore, and StromalScore levels than the other group, which had lower values (Figure 7B). RNA stemness score (RNAss) and DNA stemness score (DNAss) may be used to quantify tumor stemness (Malta et al., 2018; Zheng et al., 2021). The correlation analysis was carried out to determine whether the SRGS was related to tumor stem cells and the TME. The results in Figure 7C showed that the SRGS was not significantly correlated to the DNAss, but was positively correlated with RNAss, stromal score, and immune score. Moreover, we also found that most of the HLA genes were highly expressed in groups with higher SRGS scores (Figure 7D).

### 3.7 Relationship of the cellular senescence-related signature with tumor mutation burden and immunotherapy

**FIGURE 8:**
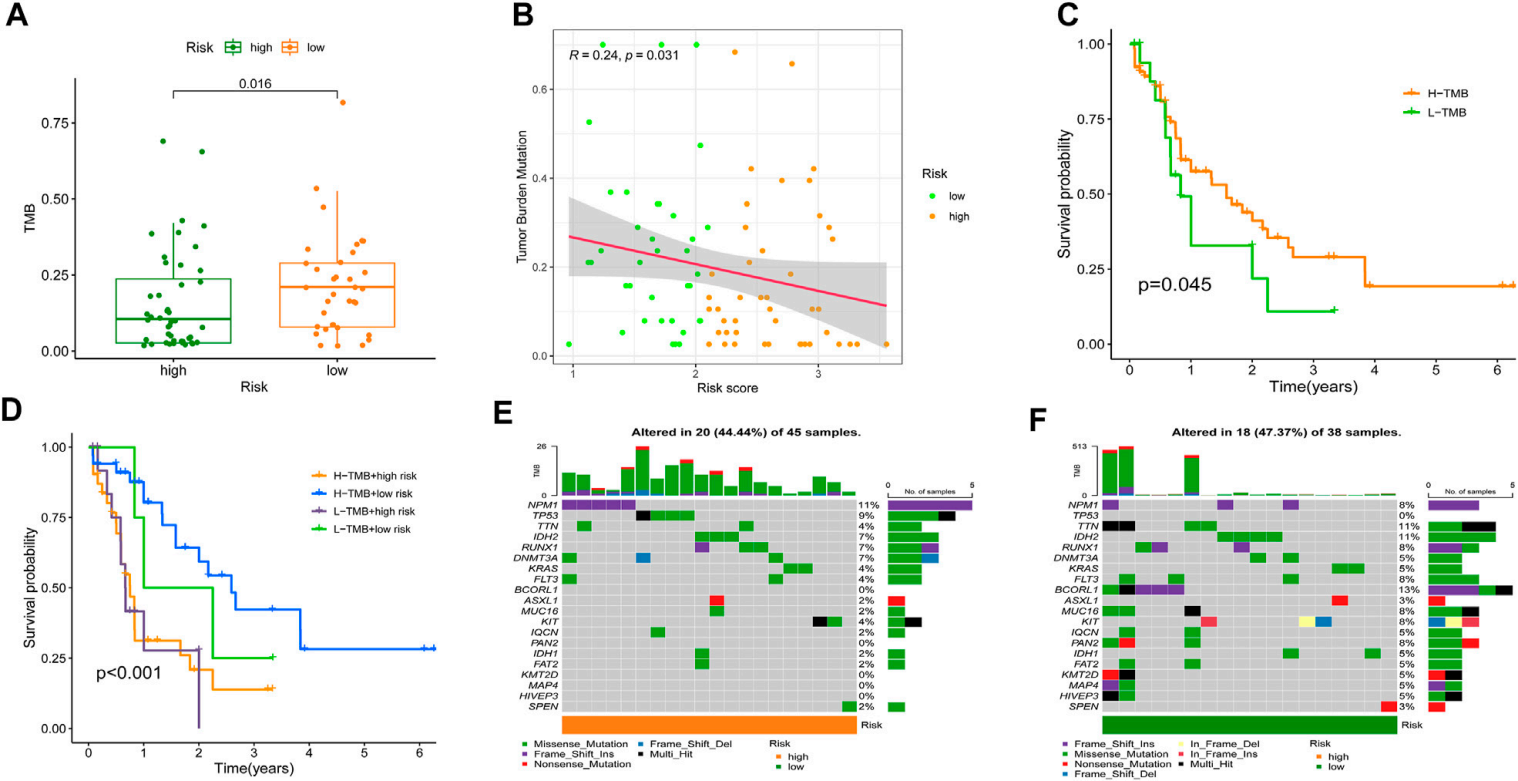
Relationship of the SRGS with TMB. **(A)** Comparison of TMB between two risk groups. **(B)** Correlation between the SRG and TMB. **(C)** Kaplan-Meier analysis on the TMB in the TCGA-AML cohort. **(D)** Kaplan-Meier analysis for the groups that stratified by combining the TMB and the SRGS score. **(E,F)** OncoPrints constructed using the high scores **(E)** and the low scores **(F)**.

Recent studies (Rizvi et al., 2015; Chan et al., 2019; McGranahan et al., 2016) have shown that a high TMB is strongly relatedtoh an excess of CD8^+^ T lymphocytes that may recognize tumor cells and elicit an antitumor immune response. For this reason, we hypothesized that TMB mamightperate as a non-negligible predictive factor of antitumor immunotherapy responsiveness. Initially, we discovered that individuals with higher SRGS scores in the TCGA-AML cohort had significantly lower TMB (Figure 8A). The correlation study of SRGS and TMB revealed a similar pattern (Figure 8B). Survival analysis demonstrated that AML patients with higher TMB had longer OS (Figure 8C). As seen in Figure 8D, patients in the group with higher SRGS scores had unfavorable OS regardless of TMB level, which indicated that SRGS in combination with TMB might serve as a potential and prognostic predictor for AML patients’ clinical outcomes. In addition, the distribution of gene mutations in both the high- and low-risk score subcategories was investigated and shown graphically. The entire landscape of somatic variations depicted the mutation patterns and clinical characteristics of the top 20 driver genes with the most common mutation (Figures 8E,F). These results may shed new light on the inherent relationship between cellular senescence and somatic variations in AML immunotherapy.

**FIGURE 9:**
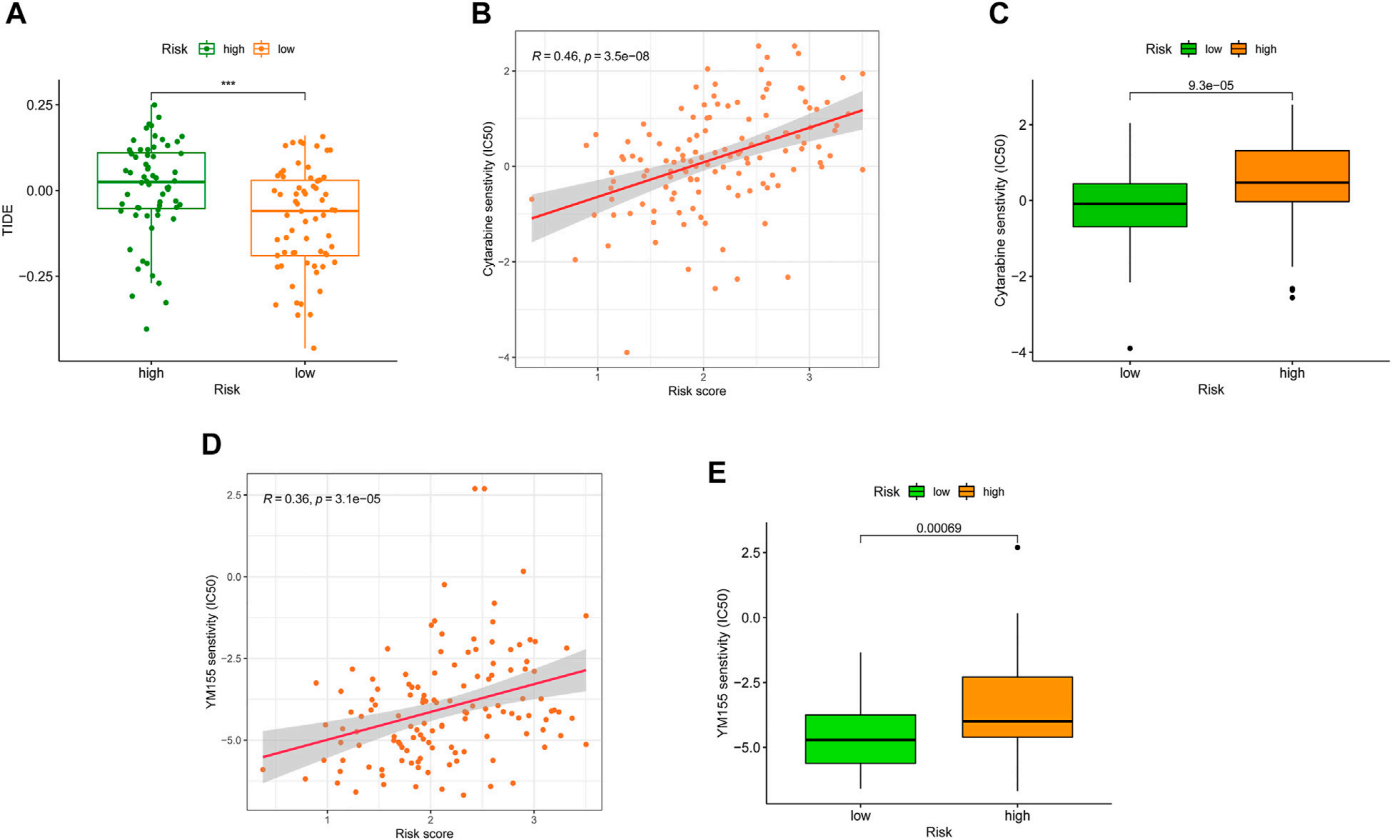
Correlation between SRGS and immunotherapy. **(A)** Distribution of TIDE scores between two TCGA-AML risk groups. **(B–E)** Correlation between the SRGS score and chemotherapeutic sensitivity.

Meanwhile, growing evidence shows that immune checkpoint inhibitors (ICIs) could improve the clinical outcomes of AML patients, but responses vary. Hence, accurate predictive biomarkers for AML are urgently needed. Given the association between SRGS, immune infiltration, and checkpoints, we analyzed the correlation between the proposed SRGS and the recognized immunotherapy predictor TIDE (Fu et al., 2020; Jiang et al., 2018). We discovered that patients with higher SRGS scores tended to achieve higher TIDE scores (Figure 9A), indicating that patients in the low-risk group may benefit from ICIs. Since the SRGS score was associated with poor prognosis in AML, the relationship between the SRGS score and chemoresistance was explored. The IC_50_ was calculated to predict the treatment response to cytarabine (Donnette et al., 2021) and YM155 (de Necochea-Campion et al., 2015). Lower SRGS AML samples were more sensitive to cytarabine (Figures 9B,C), widely used in treating patients with AML. Meanwhile, YM155, an effective survivin inhibitor, seemed to have a better curative effect on AML patients with lower SRGS scores (Figures 9D,E).

To further confirm the results of drug-relevant analysis, we investigated the expression of SRGs in the proposed signature in U937 cells treated with cytarabine or YM155. Firstly, we tested cell viability following exposure to cytarabine or YM155 of AML cell lines. As shown in Supplementary Figures S2A,S2D, cells were exposed to cytarabine or YM155 for 48 h, prior to cell viability analysis via CCK-8. As predicted, our results revealed that cytarabine or YM155 treatment could induce apoptosis (Supplementary Figures S2B,S2E). Further experiments demonstrated that KDM5B and SMURF2 were positively related to the apoptosis of AML cells. At the same time, MAP4K1, G6PD, CDK18, SOCS1, ETS2, and AKR1B1 showed opposite trends (Supplementary Figures S2C,S2F), suggesting that KDM5B and SMURF2 might be protective factors for AML patients. These results confirmed that eight genes in the SRGS may play essential roles in the efficacy of cytarabine or YM155 in AML patients, similar to the previous analysis (Figure 9).

## 4 Discussion

Senescence is a complicated process, which involves cell-autonomous and paracrine effects, and is found to have a substantial influence on the microenvironment (Hernandez-Segura et al., 2018; Lasry and Ben-Neriah.,2015). There is growing evidence that senescent cells may be removed by an immunological response triggered by SASP that includes both innate and adaptive immunity (Schneider et al., 2021). It is conceivable that the SASP has numerous beneficial short-term roles. However, in the immunosuppressive environment of cancer, these capabilities may turn deleterious and encourage tumor formation over time (Basisty et al., 2020; Birch and Gil., 2020). However, it has not been described how senescent cells interact with immune infiltration in tumors or their utility in assessing the immune infiltration of malignancies. Determining if senescence molecular factors remodel TMEs and whether this transformation has any consequences for the clinical prognosis and therapeutic response of AML patients will need modeling AML. Importantly, understanding how cellular senescence affects the TME might pave the way for senolytic medicines that successfully ameliorate the immunosuppressive environment (van Deursen, 2019).

In this study, we explored the expression patterns of SRGs, as well as their predictive values, effects on the TME, and drug sensitivity in AML. In detail, we constructed a novel prediction model named SRGS. Then, the predictive value of the SRGS was well validated in the TCGA-AML set and an external public GEO dataset. We also explored the features of the TME in AML patients with different SRGS scores, which included immune cell distribution and the activities of the inflammatory response. Significantly, we recognized distinct SASP influencing TME remodeling as possible immune evasion and tumor growth pathways. In addition, we discovered that the SRGS was an independent predictor for AML patients when combined with immune checkpoints or TMB.

This is one of the first studies to analyze SRGs’ expression patterns and discover their prognostic values via utilizing the TCGA and GEO datasets. Six highly upregulated and two considerably downregulated genes were found and included in the SRGS proposed in this work. Intriguingly, these hallmark genes have been identified as regulators of cellular senescence in a variety of malignancies and play crucial roles in tumor formation (Ao et al., 2017; Francica et al., 2016). The ectopic expression of KDM5B suppressed AML growth (Ren et al., 2022). MAP4K1 has been found not only can regulate drug resistance but also can independently predict AML prognosis (Ling et al., 2021). The acetylation regulation of G6PD is also found to be involved in the metabolic reprogramming of AML (Xu et al., 2016). The SOCS1’s ubiquitin-mediated degradation plays a vital role in the genesis of AML (Wu et al., 2018). The expression of ETS2 is linked to the biology of AML in not only DS but also non-DS children (Ge et al., 2008). These published efforts provide evidence that further support the SRGS has the potential to predict AML prognosis.

As the significance of cellular senescence in cancer is mainly unexplored, it is essential to get a deeper understanding of the relationships among cancer, senescence, and the immunological milieu. To yet, however, the effect of cellular senescence on the tumor immune infiltration has been inadequately investigated, as well as whether or not this might influence the therapeutic response to ICIs. By undertaking a comprehensive assessment, we showed that SRGs might have significant impacts on the composition and location of the tumor immune cell infiltration. Moreover, we found that the SRGS score was negatively correlated with the infiltration levels of activated mast cells, resting mast cells, follicular T helper cells, cancer-associated fibroblasts, NK cells, and regulatory T cells. In contrast, it was positively correlated with the infiltration levels of M2 macrophages, B cells, monocytes, and myeloid dendritic cells in AML. This data revealed that individuals with higher SRGS scores might have a tumor microenvironment that is immunosuppressive, preventing the immune system from eliminating tumor cells. Then, to further investigate the processes of immunological remodeling caused by the rising number of senescent cells, we discovered that SASP changes might influence TME establishment, which leads to immune evasion and promotes tumor progression. The group with higher SRGS scores demonstrated increases in inflammatory regulators, such as IL-16 and CXCLs; growth factors, including ANG, AREG, EREG, FGF7, and HGF; receptors, including ICAMs, PLAUR, and TNFRSF11B; and proteases, including CTSB and SERPINE1. These variables may influence the recruitment of immune cells and promote tumor growth (Basisty et al., 2020; Hari and Acosta., 2017; Lau and David., 2019). In addition, immunological remodeling associated with cellular senescence may explain the reduced efficiency of immune checkpoint blockade. Intriguingly, PD-1, CTLA-4, and LAG3, three fatigued T cell markers, were abnormally elevated in AML samples with higher SRGS scores, showing that T cells might become more hypofunctional and hyporesponsive as the increase of senescence. These results may also explain why elderly individuals have a lower immunotherapy response rate.

Consequently, our results have clear clinical value. On the one hand, individuals with low SRGS scores had considerably longer longevity, indicating that high-risk AML patients should get more frequent monitoring and appropriate treatments. In contrast, given that only a subset of patients may get long-term advantages from ICIs, we want more precise biomarkers with therapeutic usefulness. The created SRGS may be used as a prognosis tool as well as a guide for customized immunotherapy. In addition, small compounds targeting SRGs have been discovered and have exhibited anticancer potential *in vitro* and *in vivo* (Gormally et al., 2014; Li et al., 2020; Polson et al., 2018). And these results demonstrate the potential for future therapeutic uses of these drugs. In addition, we hypothesize that reducing inflammation associated with cellular senescence by targeting particular inflammatory mediators may have a favorable impact on the treatment of cancer. A novel class of medications known as senolytic drugs has garnered significant interes,t and accumulating preclinical and clinical evidence points to its potential significance in conjunction with immunotherapy. Thus, this class of medications may have far-reaching consequences (Kolb et al., 2021; Prasanna et al., 2021; Waltenberger et al., 2018).

Despite the fact that our research indicated the advantages of immunotherapy and the prognosis for AML, it still had significant drawbacks. First, the eight-gene risk model was created and verified using a publicly available dataset; hence, external validation in multicenter cohorts is required. Second, prospective clinical studies are required to confirm the relevance of our study findings to AML patients undergoing immunotherapy. Third, *in vivo* and *in vitro* studies of the mechanisms through which SRGs remodel the TME in AML are necessary. In addition, further research is required to demonstrate how the aging TME contributes to AML development. The early assessment of the processes behind the connection between SRGs and a worse response to ICIs needs to be clarified by utilizing fundamental investigations.

In conclusion, our work discovered and validated an SRGS with independent prognostic value for AML patients. Notably, the SRGS was strongly related to the immune cell infiltration levels and was implicated in the control of the immunological milieu in AML by SASP. At the end of this study, we characterized the complex interaction between the SRGS and immune checkpoint genes in AML. Meanwhile, we suggested the potential usage of the SRGS in combination with specific checkpoints as the predictive biomarkers of ICI response, which enabled a more accurate selection for AML patients who might benefit from ICI immunotherapy. Consequently, identifying SRGs influencing tumor immune responses and further investigating their regulatory mechanisms should aid in the risk classification and present intriguing targets which could enhance the immunotherapeutic response of AML.

## Data Availability

The datasets presented in this study can be found in online repositories. The names of the repository/repositories and accession number(s) can be found in the article/Supplementary Material.
