# Supplementary figures and images for: Comprehensive analysis for cellular senescence-related immunogenic characteristics and immunotherapy prediction of acute myeloid leukemia
Source: Front Pharmacol. 2022 Sep 26;13:987398. doi: 10.3389/fphar.2022.987398 (PMC9548549; doi:10.3389/fphar.2022.987398)

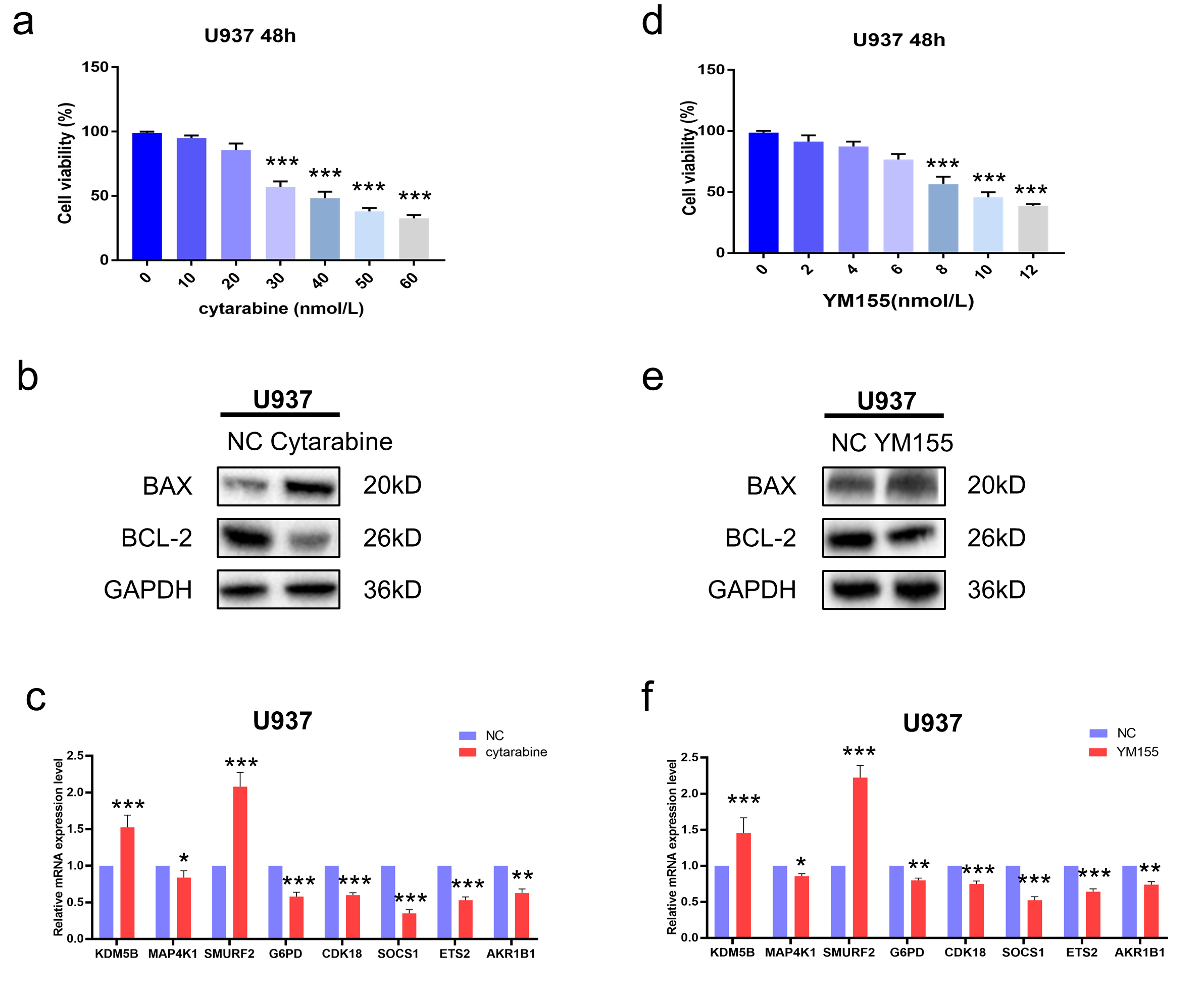

Supplement: Supplementary file 1 [file Image2.TIF]

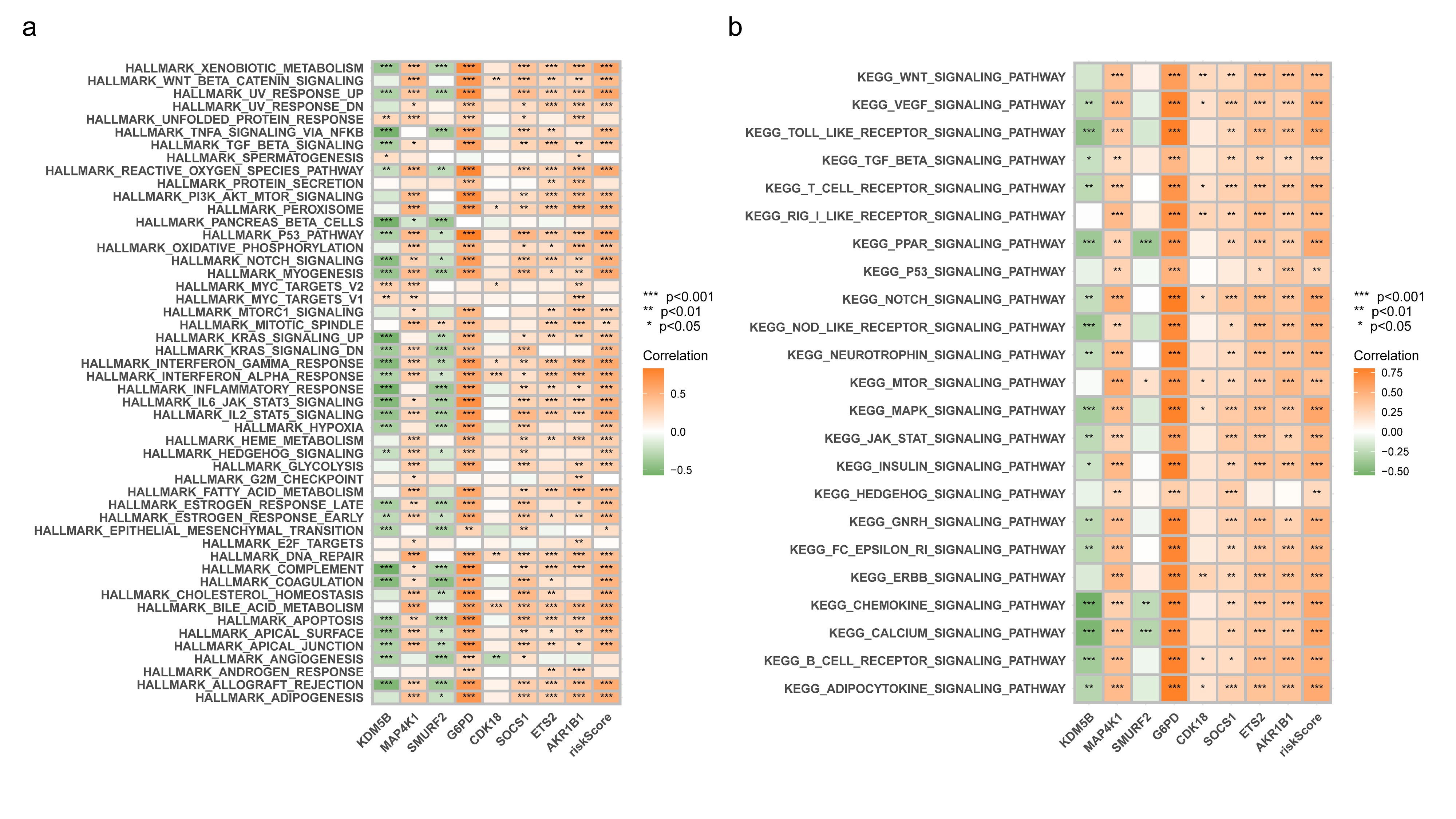

Supplement: Supplementary file 2 [file Image1.TIF]
